# Severe cumulative skin toxicity during toripalimab combined with vemurafenib following toripalimab alone

**DOI:** 10.1515/biol-2022-0606

**Published:** 2023-05-18

**Authors:** Hong Zhou, Xiao-Feng Li, Mu-Jin Chen, Li-Li Cai

**Affiliations:** Departmentof Pharmacy, Fujian Maternity and Child Health Hospital, Fujian, Fuzhou, China; Departmentof Pharmacy, First Hospital of Quanzhou Affiliated to Fujian Medical University, Fujian, Quanzhou, China; Departmentof Oncology, First Hospital of Quanzhou Affiliated to Fujian Medical University, Fujian, Quanzhou, China

## Dear Editor

Targeted therapy and PD-1/L1 inhibitors are the main treatments for BRAF V600-mutated melanomas [1,2]. Recently, PD-1 inhibitors for melanoma with toripalimab have been developed [3], and vemurafenib is a synthetic oral BRAF inhibitor. Previously, skin toxicity appeared to be more severe in patients receiving vemurafenib following anti-PD-1 therapy [4,5]. Here we describe a rare and severe cumulative dermatologic toxicity (grade 4) that developed in a patient with metastatic cutaneous melanoma during toripalimab combined with vemurafenib following toripalimab alone.

**Figure 1:**
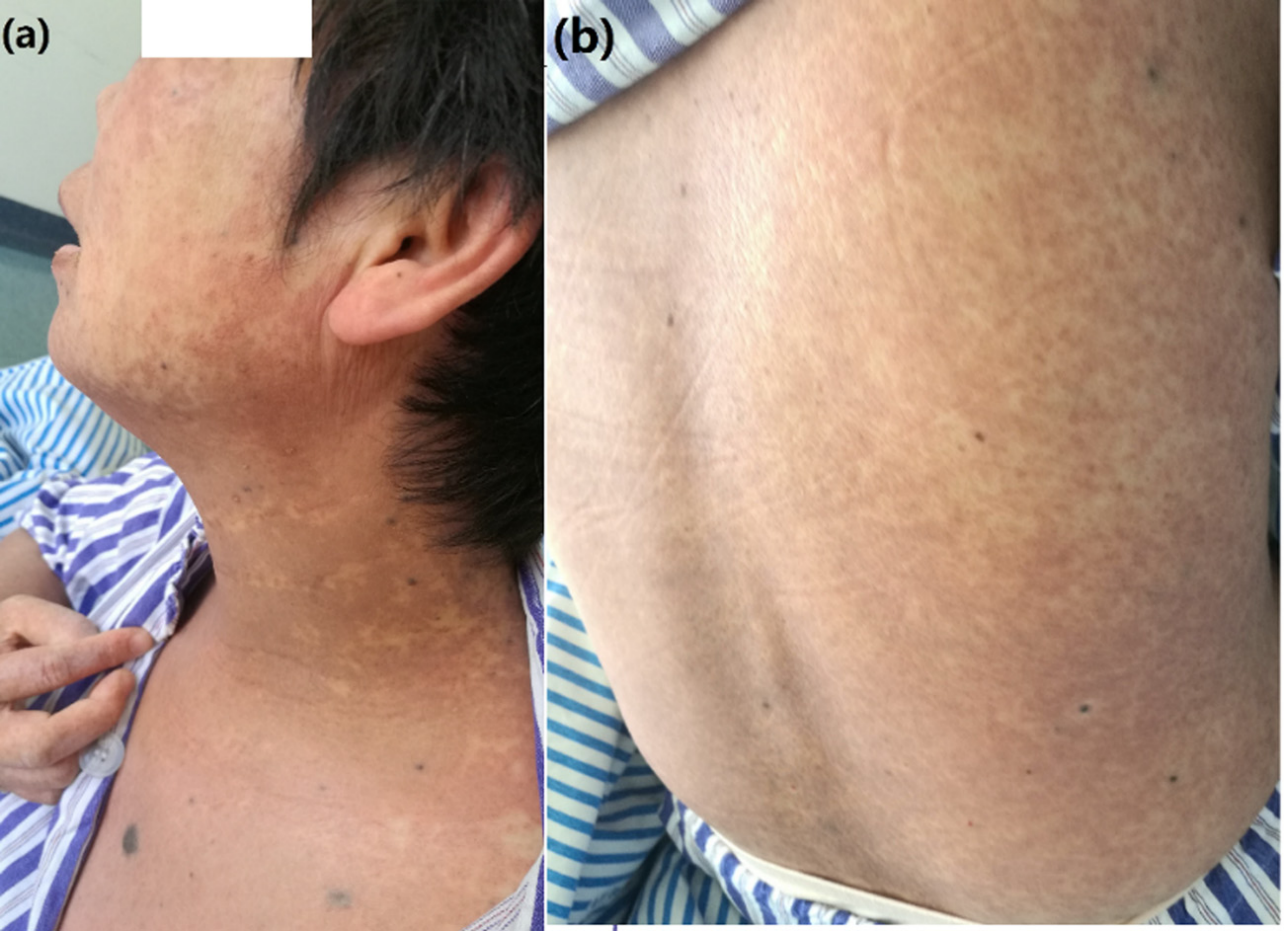
(a) Patient with disseminated cutaneous eruption of dark red pruritic macules throughout the body. (b) Non-diffuse red macules without pruritus almost entirebody after vemurafenib rechallenge.

A 52-year-old Chinese woman with abdominal cutaneous melanoma presented with multiple metastases. The patient received 3 mg/kg of toripalimab intravenously every 2 weeks, for four cycles and initiated toripalimab combined with vemurafenib 960 mg twice orally after detecting the BRAF V600E mutation. After two months, the patient developed a disseminated cutaneous eruption with red pruritic macules throughout the body, which after 2 days, became confluent with pruritus, burning, tightness, and tenderness (Figure 1a). The patient had small vesicles on the palms and wrists, mild oral mucosal erosions, and mild edema on the lower eyelids. The patient felt extreme pain. After urgent dermatology consultation, toripalimab and vemurafenib were withheld, and prednisone 2 mg/kg/day was initiated. After 1 week, the symptoms responded to corticosteroid therapy and the skin on the back and neck showed epidermal detachment, and prednisone was reduced gradually to 40 mg. However, the metastatic lesions relapsed, and the treatment was restarted. Since this was a rare and severe skin toxicity (grade 4), toripalimab was discontinued permanently. We restarted vemurafenib at the standard dose, along with prednisone 40 mg/day. Symptoms of skin toxicity partially appeared again, such as red macules, tightness (Figure 1b), and tenderness with fever of 38.8°C. Vemurafenib was discontinued again, and the rash quickly resolved with prednisolone 60 mg. After 2 weeks, we cautiously attempted to resume vemurafenib at a lower dose of 480 mg twice orally with prednisolone 10 mg/day. After 2 weeks, the dose of vemurafenib was increased to 960 mg twice orally, with no further recurrence of the rash, and prednisolone was tapered to 4 mg. On follow-up, the patient’s skin symptoms did not worsen, and the dose was deemed tolerable.

Causality assessment between toripalimab/vemurafenib and the severe skin rash via Naranjo nomogram questionnaire yielded a score of 7/10 [6], which means side-effect is very probably caused by toripalimab and vemurafenib. Corticosteroids are the mainstay of treatment of most irAEs related to immunotherapy [2].

Similar cases have been reported in which a more severe rash occurred as the patient was previously treated with anti-PD-1 therapy [4,5]. The patient’s skin toxicity was observed during toripalimab combined with vemurafenib following toripalimab alone, and reappeared partly as vemurafenib was readministered. Moreover, BRAF-targeted therapy with vemurafenib increases tumor CD8 + T-cell infiltration and upregulates PD-L1 [7]. Data indicated that adding vemurafenib may increase the immunotherapy effect of anti-PDL1 agent atezolizumab by increasing PD-L1 expression. Intricate interactions occurring during sequential and combined therapies with immune checkpoints and kinase inhibitors may lead to hypersensitivity. There have been several cases of successful vemurafenib rechallenge with dose reduction and corticosteroid administration [4,5]. We reattempted twice and successfully restarted vemurafenib by gradual dose escalation with prednisolone dose tapering.

According to the National Comprehensive Cancer Network guidelines for melanoma [2], anti-PD-1 monotherapy is the preferred regimen, and combination-targeted therapy is recommended as first-line therapy if BRAF V600-activating mutation is present. Particularly, cases combining vemurafenib with anti-PD-1 therapy after receiving anti-PD-1 therapy alone will increase, similar to the one in present study. Thus, our case highlights the importance of maintaining a high level of vigilance for severe additive skin toxicity if vemurafenib is combined with toripalimab.

## References

[1] Ascierto PA, Stroyakovskiy D, Gogas H, Robert C, Lewis K, Protsenko S, et al. Overall survival with first-line atezolizumab in combination with vemurafenib and cobimetinib in BRAFV600 mutation-positive advanced melanoma (IMspire150): second interim analysis of a multicentre, randomised, phase 3 study. Lancet Oncol. 2023 Jan;24(1):33–44. 10.1016/S1470-2045(22)00687-8 36460017

[2] Swetter SM, Thompson JA, Albertini MR, Barker CA, Baumgartner J, Boland G, et al. NCCN Guidelines® Insights: Melanoma: Cutaneous, Version 2.2021. J Natl Compr Canc Netw. 2021 Apr 1;19(4):364–76. 10.6004/jnccn.2021.0018 33845460

[3] Keam SJ. Toripalimab: First global approval. Drugs. 2019;79:573–8. 10.1007/s40265-019-01076-2 30805896

[4] Naqash AR, File DM, Ziemer CM, Whang YE, Landman P, Googe PB, et al. Cutaneous adverse reactions in BRAF positive metastatic melanoma following sequential treatment with BRAF/MEK inhibitors and immune checkpoint blockade or vice versa. A single-institutional case-series. J Immunother Cancer. 2019 Jan 8;7(1):4. 10.1186/s40425-018-0475-y PMC6323838 30621779

[5] Tsuboi S, Yoshino K, Yamaguchi K, Imafuku K, Ohara K. Two cases of successful treatment for severe skin rash induced by vemurafenib following nivolumab therapy without cessation of vemurafenib. J Dermatol. 2017;44:607–8. 10.1111/1346-8138.13489 27334634

[6] Naranjo CA, Busto U, Sellers EM, Sandor P, Ruiz I, Roberts EA, et al. A method for estimating the probability of adverse drug reactions. Clin Pharmacol Ther. 1981 Aug;30(2):239–45. 10.1038/clpt.1981.154 7249508

[7] Sullivan RJ, Hamid O, Gonzalez R, Infante JR, Patel MR, Hodi FS, et al. Atezolizumab plus cobimetinib and vemurafenib in BRAF-mutated melanoma patients. Nat Med. 2019;25:929–35. 10.1038/s41591-019-0474-7 31171876

